# Reducing stress and supporting positive relations in families of young children with type 1 diabetes: A randomized controlled study for evaluating the effects of the DELFIN parenting program

**DOI:** 10.1186/1471-2431-12-152

**Published:** 2012-09-20

**Authors:** Heike Saßmann, Mira de Hair, Thomas Danne, Karin Lange

**Affiliations:** 1 Hannover Medical School, Medical Psychology OE 5430, Hannover 30625, Germany; 2 Kinderkrankenhaus auf der Bult, Hannover 30173, Germany

**Keywords:** Children, Parenting program, Type 1 diabetes, Randomized clinical trial, Behaviour modification

## Abstract

**Background:**

To assess initial efficacy and feasibility of a structured behavioural group training (DELFIN) for parents of children with diabetes type 1, in order to reduce parenting stress and to improve parenting skills.

**Methods:**

A randomized controlled study was conducted between July 2008 and September 2010, at a children’s hospital in Hannover with parents of children with type 1 diabetes (2–10 yrs) (intervention group n = 37; control group n = 28). Parenting skills, parents’ psychological burden, children’s behavioural difficulties and quality of metabolic control were assessed before, 3 months after and 12 months after participating in the training program.

**Results:**

In the intervention group parenting behaviour in conflict situations improved significantly after 3 months (Z = −3.28; p ≤ 0.001). It remained stable over 12 months (Z = −2.94; p ≤ 0.01). Depression and anxiety scores of parents decreased (Z = −1.93; p ≤ .05; Z = −2.02; p ≤ .05). Even though the outcome in the intervention group was more positive, the differences between both study arms failed to reach statistical significance. Unexpectedly parenting behaviour in the control group improved also (Z = −2.45; p ≤ .05). Anxiety as well as stress scores decreased in this group (Z = −2.02; p ≤ .05 and Z = −2.11; p ≤ .05). In both groups the initial metabolic control was good and without significant differences (A1c 7.2±0.8% vs. 7.1±0.4%; p > 0.5). It remained stable in the DELFIN group (A1c 7.1±0.8%; p > 0.5), but it increased slightly in controls (A1c 7.3±0.5%; Z = −2.79; p = .005).

**Conclusions:**

This study has brought first evidence for the efficacy and feasibility of the program. A multicentre study with a larger sample is necessary to confirm these first results.

## Background

The therapy of children with type 1 diabetes has experienced tremendous improvements in the recent past [1-5]. In the context of a modern evidence based insulin regimen, training courses for children and parents intend to prepare families for the diabetes self-management in every day life [2,6,7]. During the last two decades structured diabetes education programs have been scientifically evaluated and accredited nationwide in Germany [2]. There are two multisite evaluated training programs for German-speaking countries for children or adolescents above the age of six [8,9]. Furthermore, there is another multisite evaluated structured education program for parents of children with diabetes [10,11]. The main aim of these programs is to impart relevant treatment knowledge and age appropriate self-management skills. The effectiveness of these programs in imparting diabetes management skills and diabetes knowledge has been proved [11-14].

In spite of the progress of the insulin therapy, its metabolic outcome is limited by family conflicts, family dysfunction and parenting problems, e. g. diffusion of responsibilities [3,15-18]. Particularly parents of younger children perceive themselves to be excessively burdened [11,19,20]. After one year of diabetes duration a positive association between parenting stress and parental depressive symptoms was described by Patton and colleagues [21].

The specific parenting issues of parents with toddlers, pre- and elementary school children (temper tantrums, implementing prohibitions and rules, conflicts between siblings and the acquisition of skills) are exacerbated by the chronic disease [22]. Dealing with daily problems might become more complicated for the parents due to continuous requirements of the diabetes therapy and lack of social support.

On the other hand parents’ well-being and positive family functioning seem to be associated with better metabolic control [15,23]. The importance of a balanced, cooperative family atmosphere has been reviewed and documented particularly for adolescents and older children with diabetes [19,24,25]. In the ISPAD Consensus Guidelines Delamater stated that behavioural concepts could support a positive family atmosphere and thereby improve psychological and physical outcomes of children with type 1 diabetes [25]. Several national and international paediatric diabetes guidelines strongly recommend pedagogic and psychological support for parents of children with diabetes [2,4,25]. Concurrently randomized controlled trials on this topic are rare [26-28]. Some elements of (cognitive) behavioural based parenting trainings have been evaluated scientifically with first results being promising [29-33]. Most of these studies focused on diabetes specific situations and challenges. However, there is no structured concept including general relevant (cognitive) behavioural parenting strategies. Due to the close association between general and diabetes specific parenting problems a wider range of strategies may be necessary to reduce parenting stress and family conflicts. For parents of healthy children several training programs on successfully solving typical conflict situations have been developed and implemented worldwide [for a survey see [34]. Scientific studies and meta-analyses demonstrate the efficacy of behavioural parent trainings and behavioural therapy elements on family functioning [35-38].

On this background the DELFIN parenting program is integrating general parenting strategies and diabetes specific conflict situations. It is based on principles of behaviour therapy and includes effective strategies of other general training programs [e. g. 35–38]. These strategies are supposed to reduce parental stress, anxiety and depression as well as children’s behavioural difficulties. Thus the current study for the first time evaluates the feasibility and effectiveness of a structured behavioural parenting training for parents of toddlers, pre-school and elementary school children with type 1 diabetes. It is hypothesized that the program participation (intervention group) would induce less negative parenting behaviour in conflict situations, support positive parenting behaviour in general and reduce parents’ psychological burden (anxiety, depression and stress). In addition children’s behaviour difficulties should decrease from their parents’ perspective.

## Methods

### Study design and recruitment

A randomized controlled group design with the control group being wait-listed was established. Inclusion criteria for families were: child with type 1 diabetes aged 2–10 yrs; no further psychological interventions; ability to read and speak German, willingness to participate. A total of 109 families with a child with type 1 diabetes in this age range were cared for at the children’s hospital “Kinderkrankenhaus auf der Bult”.

Parents were recruited by parent-conferences, posters, flyers or direct contact via their medical practitioner or diabetes educator. Fifty eight families gave their contact details for a preliminary telephone interview. Of these six families were excluded because they either were in psychotherapy or were unable to speak German. Twelve families refused to participate: too long drive from the clinic (n = 3); too time consuming (n = 7); no adequate child care (n = 2). Three families could not be reached. Thirty seven families were assigned to the DELFIN intervention or the waiting-list control group by simple randomization with a 1:1 allocation ratio. Of the latter, four families refused to participate after randomization (one for unknown reason, one because the family started family-psychotherapy, two for expenditure of time).

**Figure 1:**
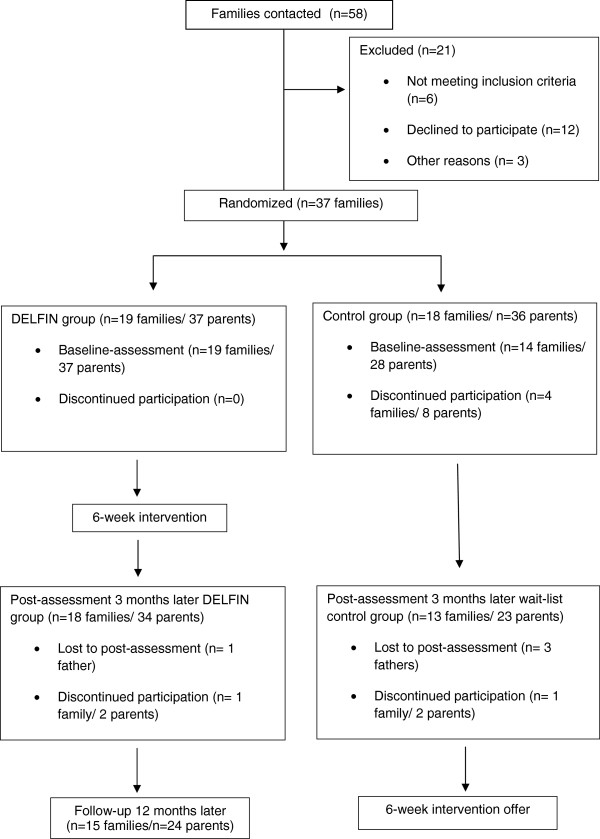
Schedule of enrollment, allocation and intervention.

Overall 65 parents (n = 33 mothers; n = 32 fathers) completed the baseline questionnaires. Fifty seven parents completed the post-assessment 3 months later and 24 parents of the intervention group completed the follow-up assessment after 12 months. The first outcome point was chosen to give the parents opportunities to establish their new skills in daily life and to be able to detect changes in A1c. The reasons for drop-out at 3 months (n = 8, 5 of them controls) were: child in psychotherapy (n = 2); severe illness of one parent (n = 2); no more interest in the study (n = 4) (Figure 1).

Parents provided assent according to approved human subject procedures. Survey data was collected through a questionnaire sent to the family’s home using a prepaid envelope. Families were provided a one-time incentive of €30 for the assessment. Medical data was taken from the routine clinical care. The study was performed according to the criteria of the Helsinki II Declaration and approved by the Ethics Committee of the Hannover Medical School (Nr. 4958).

### DELFIN intervention

**Figure 2:**
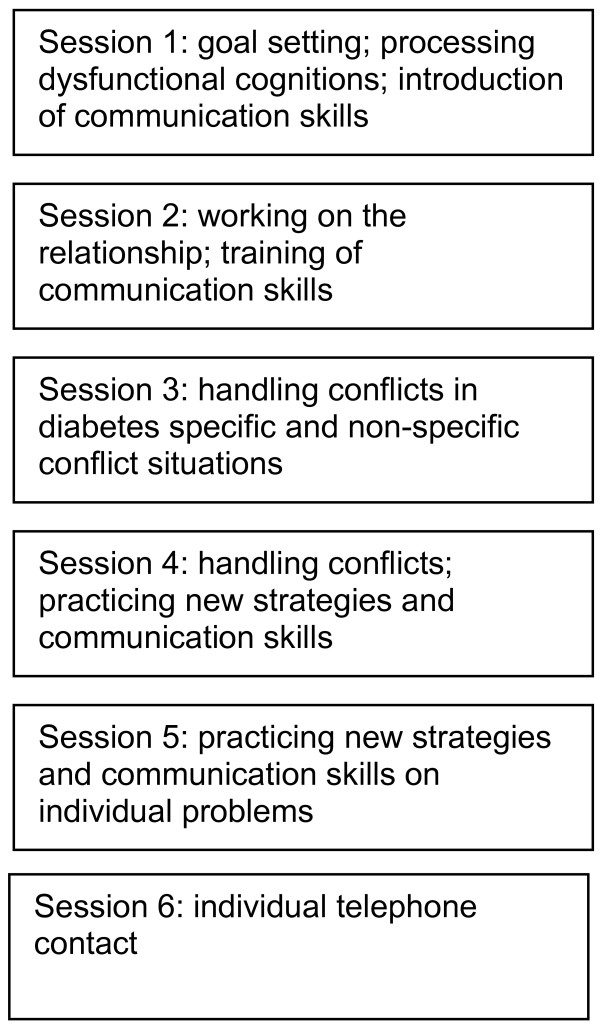
Structure of the DELFIN program.

The DELFIN program (DELFIN – **D**as **El**terntraining **f**ür Eltern von K**in**dern mit Diabetes Typ 1 (The parenting program for parents of children with diabetes type 1) was developed in addition to already existing accredited educational programs for children with type 1 diabetes and their parents. It is a structured group intervention for parents based on behavioural principles to strengthen their general and diabetes specific education competences. Parents are trained in groups with up to 7 families. They meet weekly for a 2 hours session over a period of 5 weeks and receive an individual phone contact the week after (see Figure 2). The parent training was run by an experienced psychologist.

The contents of the first session are the processing of dysfunctional cognitions, goal setting and theoretical discussion of communication skills. In the second session several strategies to work on a positive relationship with children are introduced. Parents practice communication skills in standardized role plays. Homework for these sessions includes among others the detection and substitution of dysfunctional cognitions and the phrasing of concrete and realistic goals.

In session 3 helpful parenting skills to solve typical family conflicts are developed. Sessions 4 and 5 focus on practical skill training with regard to the particular challenges of families with a chronically ill child (e. g. catheter insertion, injection, blood glucose measurement, dealing with high or low blood glucose, diabetes specific dysfunctional cognitions, feeling of guilt). These sessions also address general age specific parenting problems like temper tantrums or disobedience. Strategies are initially practiced in standardized role plays, later on in individual role plays. Weekly homework includes the transition of the new strategies into family routine.

### Measures

#### Socio-demographic data

Socio-demographic data (age, sex, diabetes duration, level of parental education, distance to the clinic) were collected via a structured questionnaire.

#### Psychological self-report inventories

The Parenting Scale [PS; [39] was used in the German version [40] to assess parent education skills in conflict situations. It consists of 35 double sided verbalized items and delivers 3 scales: overreactivity (12 items; α = .81/.81 (mothers/fathers)), laxness (11 items; α = .71/.67) and verbosity (6 items; α = .64/.58) as well as a total score (α = .81/.75). Each item is ranked on a 7 point Likert scale with the end points describing effective parenting behaviour and ineffective behaviour, respectively. The total score as well as the subscales scores are calculated via means with higher scores indicating more ineffective parenting behaviour.

The Questions to Education Behaviour [Fragen zum Erziehungsverhalten; FZEV; [40,41] form was developed to assess positive, helping and reinforcing parental behaviour. Thirteen Items were combined to a total score (α = .85/.87) via calculation of mean. The items are assessed on a 4 point scale with higher scores indicating more positive parenting behaviour.

The Depression-Anxiety-Stress Scale [DASS; [42] was used in the German version [43] and consists of 42 Items describing different psychological symptoms. It delivers 3 scales with 14 Items each: depressed mood (α = .93/.93), anxiety (α = .82/.85) and stress (α = .82/.86) as well as a total score (α = .96/.95). Items are assessed on a 4 point scale. Cumulative values are calculated with higher scores indicating higher psychological distress. Total value varies between 0 and 42 for each subscale and between 0 and 126 for the total score.

The parents’ version of the Strengths and Difficulties Questionnaire [SDQ; [44,45] is a brief behavioural questionnaire, assessing 4 areas of potential difficulties (emotional symptoms, hyperactivity, conduct and peer relationship problems). They are summed up to a total difficulties score (20 Items). Another scale assesses pro-social behaviour (5 Items). Each item is ranked on a 3 point scale with higher scores indicating more difficulties or positive behaviour, respectively. The range of the total difficulties score is 0–40 (cumulative value), the one of the pro-social behaviour score is 0–10.

Satisfaction with the DELFIN program was assessed via a structured questionnaire. Questions were rated on a 7 point Likert-scale with 1 being “not satisfied” and 7 being “very much satisfied”.

#### Biomedical data

Biomedical data was taken from the prospective medical records including A1c, frequency of acute complication (severe hypoglycaemia, DKA) and insulin regimen. A1c was measured centrally via DCA^TM^, Siemens Healthcare Diagnostics.

### Analysis

All analyses were completed using SPSS v18. Group comparisons were conducted using simple independent t-tests, Chi^2^-Tests, Wilcoxon-, Mann–Whitney-U-Tests or Kruskal-Wallis-H-Tests. In addition a general linear model was conducted for group x time analyses. Correlations between ordinal variables were conducted using Spearman’s p correlation coefficient. All results being significant at p < 0.05, unless otherwise stated.

## Results

### Socio-demographic, metabolic and psychological status at study entry

Families of the DELFIN group did not differ significantly from the controls according to age of parents, age of the index-child, number of siblings, gender of the index-child, education level of mothers or insulin delivery modality (Table 1). However, the fathers in the DELFIN group were significantly higher educated than those in the control group. All children were on intensified insulin therapy. Of those 81% (n = 27) were on CSII; the others on multiple daily injection therapy (MDI). At study entry 73% (n = 24) of the children were in good metabolic control (A1c < 7.5%), 24% (n = 8) in moderate control (A1c 7.5 - 9%) and one child was in insufficient control (A1c > 9.0%). In all but one psychological measure there were no significant differences between control and DELFIN parents. The intervention group parents scored higher on one subscale of the Parenting Scale, stating that they tend more frequently to inadequate intense reactions in parenting conflict situations than the control group parents (Z = −2.59, p = .01).

**Table 1:** Sociodemografic characteristics of the sample (control = control group; DELFIN = intervention group)

	**control**	**DELFIN**		
	**Mean (SD)**	**Mean (SD)**	**t**	**p**
age mother (yrs)	40.4 (3.8)	39.1 (3.3)	−1.04	n.s.
age father (yrs)	42.4 (6.5)	43.3 (5.5)	0.42	n.s.
age of the index child (yrs)	5.8 (1.9)	6.4 (2.3)	0.78	n.s.
number of siblings	0.79 (0.58)	0.95 (0.69)	0.75	n.s.
	N (%)	N (%)	Chi^2^ (df)	p
Education level mothers				
Certificate of secondary education (8 grades)	2 (14)	1 (5)	6.8 (3)	n.s.
General certificate of secondary education (10 grades)	6 (43)	6 (32)		
A-Level (13 grades)	6 (43)	5 (26)		
University	0 (0)	7 (37)		
Education level fathers				
Certificate of secondary education (8 grades)	1 (7)	2 (11)	9.09 (3)	**.028**
General certificate of secondary education (10 grades)	6 (43)	1 (5)		
A-Level (13 grades)	4 (29)	3 (17)		
University	3 (21)	12 (67)		
	Mean (SD)	Mean (SD)	t	p
Diabetes duration in years	2.6 (1.9)	2.6 (1.6)	−0.05	n.s.
A1C	7.1 (0.4)	7.2 (0.8)	0.82	n.s.
	N (%)	N (%)	Chi^2^ (df)	p
Insulin regimen index child				
CSII	11 (79)	16 (84)	0.23 (1)	n.s.
MDI	3 (21)	3 (16)		
rate of severe hypoglycemia last 12 month	0 (0)	2 (10)	3,22 (1)	n.s.
DKA last 12 month	0 (0)	1 (5)	1,56 (1)	n.s.

### Outcomes three months after intervention

Adverse parenting behaviour (Parenting-Scale, PS): After the intervention the difference between intervention and control group on the subscale “overreaction” was no longer existent due to improvements in the DELFIN group (Table 2). Compared to study entry the DELFIN parents improved their parenting behaviour on all 3 subscales as well as on the total score of the PS. Nevertheless, there were no significant time x group effects (all repeated-measure analyses with p > .05, with F (1/55) = 2.49 and p = .12 for the total score). Unexpectedly parents of the control group improved significantly on the subscale PS-verbosity (talking and discussing a lot with the child in conflict situations) and on the total score.

**Table 2:** Comparison control vs. DELFIN group

**Variable**	**control**				**DELFIN**			
	**baseline mean (SD)**	**post mean (SD)**	**Z**	**p ≤**	**baseline mean (SD)**	**post mean (SD)**	**Z**	**p ≤**
PS-total	3.1 (0.6)	3.0 (0.5)	−2.45	**.05**	3.4 (0.5)	2.9 (0.7)	−3.28	**.001**
PS-overreactivity	3.2 (0.9)	3.1 (0.8)	−1.20	n.s.	3.8 (0.8)	3.4 (0.9)	−2.58	**.01**
PS-laxness	2.7 (0.9)	2.5 (0.8)	−1.35	n.s.	2.6 (0.6)	2.3 (0.8)	−2.51	**.01**
PS-verbosity	3.6 (0.8)	3.2 (0.8)	−2.17	**.05**	3.6 (0.9)	3.0 (1.0)	−3.06	**.01**
DASS-total	21.9 (15.4)	17.3 (10.3)	−2.10	**.05**	25.5 (15.2)	20.0 (13.4)	−1.93	**.05**
DASS-depression	5.2 (5.1)	4.1 (4.1)	−0.96	n.s.	6.2 (5.8)	4.7 (5.4)	−1.93	**.05**
DASS-anxiety	4.9 (4.4)	3.1 (2.8)	−2.02	**.05**	4.5 (4.6)	2.8 (2.6)	−2.02	**.05**
DASS-stress	11.8 (6.5)	10.0 (5.2)	−2.11	**.05**	14.8 (7.6)	12.4 (6.9)	−1.05	n.s.
FZEV-positive parenting behavior	1.9 (0.4)	1.9 (0.4)	−1.09	n.s.	1.9 (0.4)	1.9 (0.4)	−0.22	n.s.
SDQ-total difficulties score	8.3 (4.4)	7.8 (4.1)	−1.31	n.s.	8.3 (4.8)	6.9(3.9)	−1.85	n.s.
Scores over the clinical cut-points	n = 3	n = 2	Chi^2^ = 14.6	**.01**	n = 7	n = 2	Chi^2^ = 9.92	**.01**
SDQ-prosocial behaviour	7.4 (1.9)	7.3 (1.9)	−0.73	n.s.	7.3 (1.6)	7.7(1.5)	−1.92	n.s.

Parents’ psychological distress (Depression-Anxiety-Stress-Scale, DASS): Psychological distress (total score, depression and anxiety scale) decreased significantly in the DELFIN group (Table 2). There were no significant time x group effects (all analyses from general linear models with p > .05 and F (1/55) = .02, p = .88 for the total score). Unexpectedly in the control group a significant reduction of anxiety, stress and of the total DASS-score was observed.

Positive parenting behaviour (Questions to Education Behaviour, FZEV): There were no significant differences regarding positive parenting behaviour (FZEV) before and after the training in both study groups. Also there were no differences between the groups (F (1/55) = .97, p = .33).

Strength and difficulties of the child (Strengths and Difficulties Questionnaire, SDQ): The psychological wellbeing of the children improved slightly in the DELFIN group, but this difference did not reach statistical significance neither within nor in between the two groups (F (1/55) = .05, p = .82 for the difficulties score and F (1/55) = .23, p = .64 for the pro-social behaviour score). However, the number of children above the cut-off for clinical problems decreased significantly in the DELFIN group (Table 2).

Metabolic control (A1c): While the good quality of control was stable after 3 months in the DELFIN group (7.2±0.8% vs. 7.1±0.8%, Z = −1.19, p > .05), the mean A1c increased significantly in the control group (7.1±0.4% vs. 7.3±0.5%, Z = −2.79, p ≤ 0.005). The interaction was significant in the group x time analysis (F (1/32) = 5.3, p = .029). This result has to be discussed with care due to the overall good metabolic control of the majority of children and the small sample size.

Effect sizes were calculated for the control and the DELFIN group (Table 3). They were moderate to high for the reduction of negative parenting behaviour in the intervention group. Comparing parenting behaviour (PS-Parenting Scale) and parent assessment of their child (SDQ) the effect sizes were higher in the intervention group than in the control group. For parents’ stress, depression and anxiety small effect sizes were observed in both study arms.

**Table 3:** Effect sizes (d) for control and DELFIN group

**Variable**	**control**			**DELFIN**		
	**baseline mean (SD)**	**post mean (SD)**	**effect size d**	**baseline mean (SD)**	**post mean (SD)**	**effect size d**
PS-total	3.1 (0.6)	3.0 (0.5)	.18	3.4 (0.5)	2.9 (0.7)	.84
PS-overreactivity	3.2 (0.9)	3.1 (0.8)	.12	3.8 (0.8)	3.4 (0.9)	.48
PS-laxness	2.7 (0.9)	2.5 (0.8)	.24	2.6 (0.6)	2.3 (0.8)	.43
PS-verbosity	3.6 (0.8)	3.2 (0.8)	.51	3.6 (0.9)	3.0 (1.0)	.64
DASS-total	21.9 (15.4)	17.3 (10.3)	.35	25.5 (15.2)	20.0 (13.4)	.39
DASS-depression	5.2 (5.1)	4.1 (4.1)	.24	6.2 (5.8)	4.7 (5.4)	.27
DASS-anxiety	4.9 (4.4)	3.1 (2.8)	.49	4.5 (4.6)	2.8 (2.6)	.46
DASS-stress	11.8 (6.5)	10.0 (5.2)	.31	14.8 (7.6)	12.4 (6.9)	.33
FZEV-positive parenting behaviour	1.9 (0.4)	1.9 (0.4)	.00	1.9 (0.4)	1.9 (0.4)	.00
SDQ-total difficulties score	8.3 (4.4)	7.8 (4.1)	.12	8.3 (4.8)	6.9(3.9)	.32
SDQ-prosocial behaviour	7.4 (1.9)	7.3 (1.9)	.05	7.3 (1.6)	7.7(1.5)	.26

### Outcomes at 12 months follow-up

**Figure 3:**
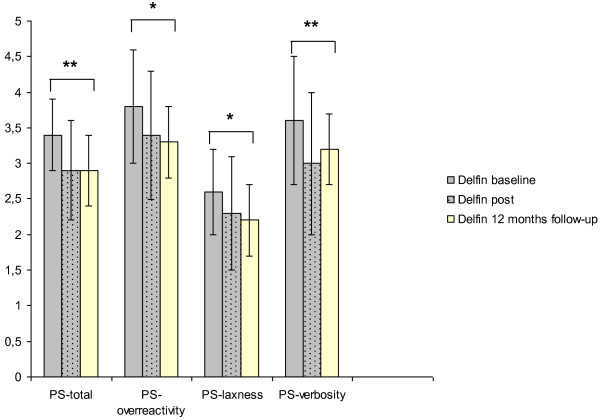
Parenting skills of DELFIN parents baseline, 3 months after the intervention and at 12 months follow-up.

The improvement in parenting behaviour in conflict situations (PS-Scale) was stable after 12 months in the DELFIN group (Figure 3). Compared to baseline measures adverse parenting behaviour decreased significantly for the total score as well as for all 3 subscales (PS-total Z = −2.9; p ≤ 0.01; PS-overreactivity Z = −2.2; p ≤ 0.05; PS-laxness Z = −2.0; p ≤ 0.05; PS-verbosity Z = −2.4; p ≤ 0.01). After 12 months there were no significant differences compared to the measures at 3 months on the PS-Scale and all other psychological measures (all p > .05).

### Associations between parents’ wellbeing and parenting skills

For the parents of the DELFIN group Spearman-correlations between the self-reporting measures are summarized in Table 4. Positive parenting behaviour (FZEV) was negatively associated with depression (r = −.37, p = .024) and with the tendency to accommodate the child in conflict parenting situations (EFB-laxness; r = −.43, p = .007). Parents’ psychological distress was positively associated with negative parenting behaviour (r = .37, p = .23). Particularly, the tendencies to back down and to overreact and exaggerate in conflict situations were associated with higher psychological burden (Table 4).

**Table 4:** Spearman-correlations for the intervention group (n = 37)

	**DASS-total**	**DASS-depr.**	**DASS-anxiet.**	**DASS-stress**	**EFB-total**	**EFB-overr.**	**EFB-laxn.**	**EFB-verb.**	**SDQ**	**A1C**
	Rho (p)	Rho (p)	Rho (p)	Rho (p)	Rho (p)	Rho (p)	Rho (p)	Rho (p)	Rho (p)	Rho (p)
FZEV-pos. behav.	-.084 (.620)	**-.37 (.024)**	-.01 (.956)	.04 (.815)	-.30 (.069)	-.27 (.106)	**-.43 (.007)**	-.04 (.797)	.26 (.124)	**-.27 (.034)**
DASS-total					**.37 (.023)**	**.51 (.001)**	**.33 (.049)**	-.01 (.971)	.21 (.223)	.14 (.279)
DASS-depr.					.30 (.071)	**.40 (.015)**	**.39 (.018)**	.017 (.919)	.07 (.664)	.14 (.264)
DASS-anxiet.					.28 (.089)	**.43 (.009)**	.28 (.089)	.09 (.609)	.26 (.125)	.05 (.711)
DASS-stress					**.33 (.043)**	**.48 (.003)**	.25 (.142)	-.04 (.821)	.18 (.298)	.10 (.443)

### Satisfaction with the DELFIN program

Twenty four parents completed the assessment on satisfaction with DELFIN program. Of them 22 were satisfied/very satisfied with the program (point 6–7) and 20 were satisfied/very satisfied with the support given by the trainer. The majority of parents evaluated all 5 sessions as comparably important (n = 13). Fourteen of the 19 families of the DELFIN group attended all 5 group sessions. Only 5 missed one group session (due to time strains, illness or lack of babysitter). Considering an average distance of 43.8 km (min 0.5 km - max 144.5 km) to the clinic this supports high intervention fidelity of the participants. Each family attended the individual telephone contact (session 6). All participants were regularly prepared for the sessions and had done their practical trainings (homework). In order to support intervention receipt, homework interviews were carried out with each parent at the beginning of the sessions. Implementation of new strategies to solve family conflict situations were reported by all participants. Individual situations were analyzed and suitable solutions were worked out, if problems occurred. Parents were asked to try out new solutions within the next week.

## Discussion

The randomized controlled pilot study assessed the effectiveness of a behavioural training program especially for parents of young children with type 1 diabetes. The results point to improved parenting skills and slightly reduced parenting stress. The pilot study on this psychological-based concept supports the feasibility of the parenting program. The first data provide important information for conducting a larger multisite clinical trial to confirm the efficacy of the program.

### Recruitment, sample and participation

The high expenditure of time was the main reason for originally interested families not to participate in the program. Therefore future studies should consider alternative methods of offering such a program since the long drive to the clinic, excessive burden due to the time investment or the lack of care for the children were the most common reasons for many families not to participate in the study. Alternatively the intervention could be delivered during routine clinic visits as successfully practiced with other interventions [46]. Short-term-interventions like seminars on weekends, specific short interventions dealing with one topic or internet-based sessions may be reasonable concepts to increase the attendance. On the other hand, more than half of the parents rated all 5 group sessions as comparably important, and many of them required longer sessions. A reduction of intervention time would also mean less information and exercise for the parents and thus probably reduced efficacy of the program.

Monaghan et al. [32] developed a brief telephone-based supportive intervention for parents, which is a very economic way to run a program. The cognitive-behavioural therapy elements of the DELFIN program (e. g. dysfunctional cognitions) may be delivered via telephone contact to reduce the number of group sessions. But the important interaction between different parents on this topic would get lost. The same may be the case for the main part of the program, the behavioural therapy elements and the practical training of parental behaviour.

In addition to more flexibility in delivering and recruiting, a higher homogeneity of age in the groups could further increase the efficacy of the training. From practical experience during the sessions it has become obvious that too high heterogeneity of the children’s age is challenging for the thematic organization of the courses. This study included families with children between 2 to 10 years of age. However, the every day conflicts of parents caring for toddlers, pre-school-children or primary school children differ considerably. Due to these age-specific challenges, different educational strategies become more important and have to be customized for each subgroup. Furthermore, there are various diabetes specific conflicts depending on the cognitive developmental status of the child and its ability for self-management. Parents of pre-school-children for instance are responsible for the entire therapy and the continuous supervision. In contrast parents of older children often have to deal with insufficient adherence and autonomy conflicts.

### Eligibility and performance of outcome measures

The measurements adopted in this study can be considered appropriate. In their oral feedback parents rated the assessment form as acceptable and easy to understand. In a larger trial it would be desirable to include behavioural observations, assessment by (day care) teachers and an interview with the parents. Observational data could be collected during a home visit. This would probably also increase the retention rate.

The participants of the DELFIN-program reported a reduction in adverse parenting behaviour and partial improvement of psychological wellbeing. Probably due to the small sample size, these effects were not significant compared to the control group. Another reason might be that slight reductions in psychological distress, adverse parenting behaviour and behavioural difficulties of the child were also seen in the control group. These findings may be based on the Hawthorne-effect or were probably accidental. On the other hand, parenting behaviour might improve step by step without external intervention. This hypothesis contradicts findings of other authors describing negative parental behaviour as stable [38,40]. Another explanation might be a tendency of parents’ to give socially desirable answers. This problem could be accounted for by observational data in further studies.

The reduction of negative parenting behaviour was stable after 12 months in the intervention group, indicating that parents implement new strategies in their day to day behaviour on the long run. Unfortunately no changes were detected for positive parenting behaviour. Parents of children with type 1 diabetes have to spend many hours together with their child managing diabetes therapy. Due to this it’s probably difficult for them to increase their positive parenting behaviour e.g. playing or joking with the child.

### Limitations

It has to be considered that there was a self selection in this pilot sample, which might have influenced the outcome. Particularly in the control group many families refused to participate after being randomized. For them the waiting period might have been too long. Instead some of them looked for immediate psychological support and dropped out. This probably resulted in an overlap of families with a positive parenting status in the control group. Another critical point is the small sample size. A larger group of parents would allow more differentiated analysis, e. g. according to gender, participation rate, education level, child’s age and quality of metabolic control. Findings in this study are limited by conjunct observations of mothers and fathers, while other studies showed differences in parenting behaviour and outcome performance between mothers and fathers [41]. The study was performed in one German paediatric diabetes centre with an experienced multidisciplinary team caring holistically for children with diabetes and their families as standard care. Independent from this study the metabolic control of all children aged < 12 yrs is relatively good (mean centre A1c prepubertal children (n = 109) = 7.1%). In this overall positive situation it is challenging to improve the metabolic and psychological outcomes. This quite positive situation at study onset might not be representative for other pediatric diabetes units in Germany or worldwide [47].

## Conclusions

This study provides preliminary evidence that parents of children with type 1 diabetes may benefit from a parenting program focussing on specific as well as unspecific conflict situations and practical training of positive parenting strategies. There was first evidence that the DELFIN program for parents of young children with type 1 diabetes tends to improve parenting behaviour and the psychological status of the parents. The program predominantly reduced negative parenting behaviour. Due to the small sample size of this pilot study a long term multisite clinical trial is necessary to confirm these first findings. It is a great challenge to organize parent trainings for families often driving long distances to the specialized diabetes centres. To realize an adequate sample size, the program should be offered and evaluated in different settings, e.g. weekend meeting, specific short interventions or internet based sessions. Homogeneous age groups could help to reduce the required time for group sessions. In addition to parents’ self-reports as outcome parameters, observational data on their behaviour should be included.

Heike Saßmann is the Guarantor of this work and, as such, had full access to all of the data in the study and takes responsibility for the integrity of the data and the accuracy of the data analysis.

## Competing interests

The author(s) declare that they have no competing interests.

## Authors’ contributions

H.S., K.L., M.dH. wrote the manuscript, H.S. and M.dH. analysed the data, H.S., M.dH., K.L. and T.D. contributed to data collection and/or commented on the paper. All authors read and approved the final manuscript.

## Pre-publication history

The pre-publication history for this paper can be accessed here:

http://www.biomedcentral.com/1471-2431/12/152/prepub
